# Novel concept microarray enabling PCR and multistep reactions through pipette-free aperture-to-aperture parallel transfer

**DOI:** 10.1186/1472-6750-10-71

**Published:** 2010-10-06

**Authors:** Yasunori Kinoshita, Takahiro Tayama, Koichiro Kitamura, Md Salimullah, Hidekazu Uchida, Miho Suzuki, Yuzuru Husimi, Koichi Nishigaki

**Affiliations:** 1 Department of Functional Materials Science, Graduate School of Science and Engineering, Saitama University, 255 Shimo-okubo, Saitama 338-8570, Japan; 2 Janusys Corporation, #508, Saitama Industrial Technology Center, 3-12-18 Kami-Aoki, Kawaguchi, Saitama 333-0844, Japan; 3 Rational Evolutionary Design of Advanced Biomolecules, Saitama (REDS), Saitama Small Enterprise Promotion Corporation, #552, Saitama Industrial Technology Center, 3-12-18 Kami-Aoki, Kawaguchi, Saitama 333-0844, Japan; 4 City Area Program (Saitama Metropolitan Area), Saitama Small and Medium Enterprises Development Corporation, 2-3-2 Kamiochiai, Chuo-ku, Saitama City, Saitama 338-0001, Japan; 5 Department of Electrical and Electronic System, Graduate School of Science and Engineering, Saitama University, 255 Shimo-okubo, Saitama 338-8570, Japan

## Abstract

**Background:**

The microarray has contributed to developing the omic analysis. However, as it depends basically on the surface reaction, it is hard to perform bulk reactions and sequential multistep reactions. On the other hand, the popular microplate technology, which has a great merit of being able to perform parallel multistep reactions, has come to its limit in increasing the number of wells (currently, up to 9600) and reducing the volume to deal with due to the difficulty in operations.

**Results:**

Here, we report a novel microarray technology which enables us to explore advanced applications, termed *microarray-with-manageable volumes *(MMV). The technical essence is in the pipette-free direct parallel transfer from well to well performed by centrifugation, evading the evaporation and adsorption-losses during handling. By developing the MMV plate, accompanying devices and techniques, generation of multiple conditions (256 kinds) and performance of parallel multistep reactions, including PCR and *in vitro tr*anslation reactions, have been made possible. These were demonstrated by applying the MMV technology to searching lysozyme-crystallizing conditions and selecting peptides aimed for Aβ-binding or cathepsin E-inhibition.

**Conclusions:**

With the introduction of a novel concept microarray (MMV) technology, parallel and multistep reactions in sub-μL scale have become possible.

## Background

Introduction of the microarray has brought about a revolutionary change in biological studies, leading to the development of omic world studies: genome, transcriptome, proteome, and others [1,2]. The microarray technology has a wide range of applications and is still developing. Among the most common applications, there are cDNA and oligonucleotide chips [3,4] and expression array of proteins [5,6]. Much advanced applications are appearing as the living protein chip [7,8]. In essence, the current microarray technology is based on the surface reaction allocated at each spot. Therefore, in a microarray experiment, the elements of reactants (*e.g*., a set of mRNAs expressed in a particular cell) can be sorted and arrayed by a set of counter-reactants (*e.g*., oligonucleotides of different sequences) at a single operation, saving laborious experiments and costly reagents. For the researcher's convenience, microarrays which deal with a high density of information need to be supplied commercially due to the requirement of elaborate apparatuses and software. Obviously, most of the data obtained by the microarray technology could not have been addressed by any other method, featuring the exceptional power of this technology.

Nevertheless, the current microarray technology still leaves room to be developed. One of the emerging desires to it is the ability of multistep operations and reactions: *e.g.*, if an mRNA binding to a particular oligonucleotide could be translated into a protein *in situ *and then could be monitored about the function of the product (protein), functional analyses of proteins would be much boosted up. On the other hand, in an *in vitro *evolution study, a huge number of clones (more than 10^3-4^) need to be screened at once, meaning that each clone can be in a very small amount (less than μL) and highly parallel (more than thousands) and multistep operations are required [9-12]. In other words, the conventional micro-plate (96- and 384-wells) technology, though it allows us to operate in multisteps, requires not a tiny amount of reagents (more than 10 μL or so) if we consider the high degree of parallelism. Therefore, technologies dealing with less than μL aliquots have been pursued. Although there are not a few studies on dealing with sub-μL solutions [13-15], they are basically microfluidics approaches and are currently limited in the degree of parallelism and the number of possible reaction steps [16]. This may be because they are adopting, in a sense, a closed system so as to prevent from evaporation-loss of a tiny volume of sample, leading to rather elaborate and complicated systems. Another approach to earn the multiplicity and smallness of samples is the beads-based one [17] and the essence of it has been successfully adopted in the machine of giga-base (next-generation) sequencers [18]. However, in this approach, there is a difficult problem to cope with: multi-parallel and multistep reactions require the compartmentation of beads. This has already been challenged by some scientists [19]. We made a quite different, unique approach for this challenge: open well type compartmentation.

**Figure 1:**
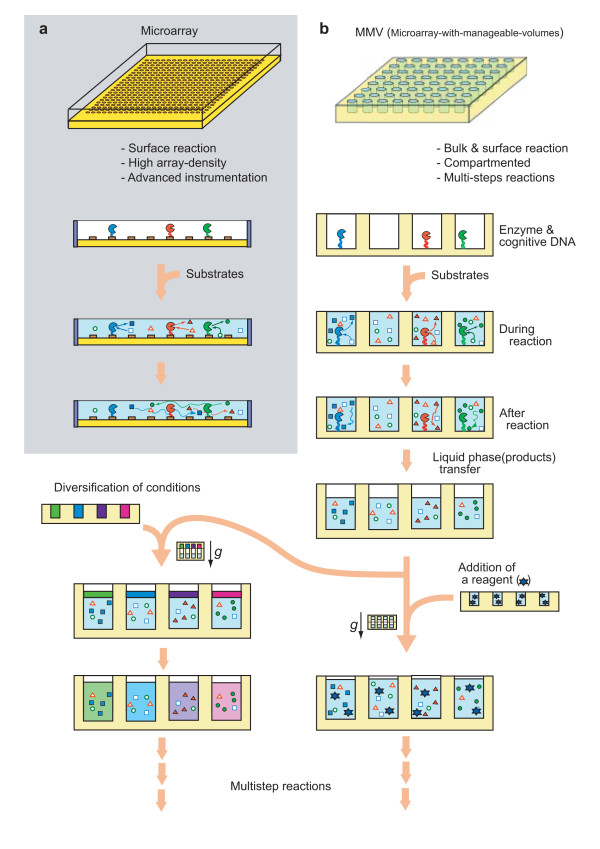
**Advantages of the MMV (*microarray-with-manageable volume*s)**. In the conventional microarray, reaction products diffuse away (Panel a) while in the MMV each reaction product can stay in the same site (well) (Panel b). Another merit of the MMV is that each well of an MMV can be treated individually, leading to parallel multistep reactions. In these experiments, the volume of an MMV well can be changed quantitatively as shown in this figure.

In this stream, we have devised a multi-well type microarray (open system) made of plastics (dry) and gel (wet) and developed its operational method through pilot experiments such as an arrayed-colony formation, a parallel monitoring of various conditions (for crystallization), and an application to multistep reactions. To develop such technologies, fabricating microarrays in-house was actually prerequisite. As is well-known, the conventional microarray technology has been developed based on the highly sophisticated photo-lithographic technology developed for the IC industry [20]. Therefore, the technological framework was well-established and rather easy to attain a high density of arrays (*e.g*., millions per square inches). Though the IC-derived technology is good at surface reactions, this technology developed for handling 'information (state)' but not 'materials (molecules)' has a limitation in being applied to a bulk reaction of molecules. One typical inconvenience is, as shown in Fig. 1, in the inability to succeed individual reaction products at each spot for the next reaction independently, thus making the parallel multistep reactions impossible. Since almost all reactions *in vitro *need to be multistep ones, a number of separate tubes (or their collective form termed as microplate) have been traditionally used with an aid of robotics, resulting in a massive system [21-23]. Currently, the number of wells per micro-plate has boosted up to almost the limit of 3456 to 9600 with increase of difficulties in operation time and throughput accuracy [24]. In this paper, a novel method depending on a wet and dry microarray system was developed, which enables parallel operations of sub-μL aliquots by overcoming difficult problems such as measuring and transferring sub-μL solutions in parallel without critical evaporation and adsorption-losses of samples. As a result, multi-different conditions were formed for crystallization of proteins and multistep reactions were successively performed in parallel beginning with PCR followed by transcription, translation, modifying enzyme reaction, and protease-activity assay. Thus, this paper reports a great potentiality of a novel concept microarray MMV for the first time.

## Results and discussion

First, we need to describe how to make and handle the novel microarray MMV and then proceed to its applications.

### Introduction of microarray with manageable volumes (MMV): how to make and operate

**Figure 2:**
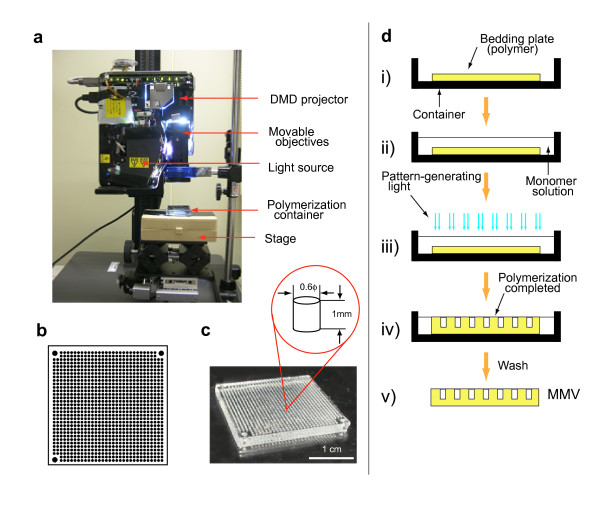
**How to generate and operate MMVs**. (a) MMV generator. (b) A projection pattern used for 1000 well type (actually 988 wells for samples and 3 guide holes). (c) An example of a plastic MMV (1024 well type). (d) Procedures for generating an MMV. Namely, i) place a bedding plate (plastics or pre-molded gel) in a container, ii) pour the monomer solution (for either plastics or gel formation), iii) irradiate the pattern-carrying light (see Fig. 2b), iv) remove the non-polymerized solution (blank) and, finally, v) get an MMV (yellow).

**Figure 3:**
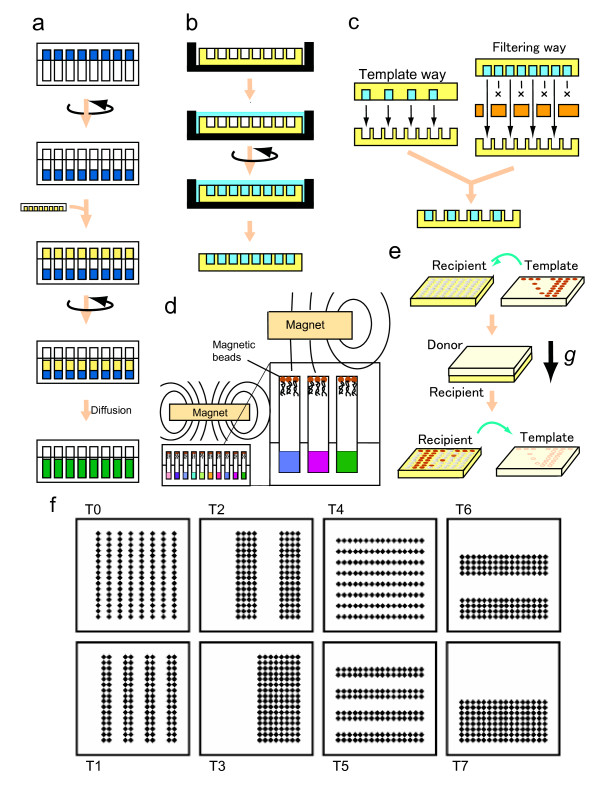
**Basic sample-handling procedures**. (a) Digital (discrete) addition of samples into MMV wells. (b) Initial filling an MMV with sample solution. Aero-bubbles are removed by centrifugation. (c) Two ways of regulatory replica-transfer: template way and filtering way. (d) Magnetic separation of beads. During the centrifugation, magnetic beads can resist against falling owing to the magnetic force. (e) Replica-transferring of samples into recipient MMV wells. (f) Template MMVs (T0~T7) used for selective introduction of samples. These 8 patterns of MMVs can generate 256 species of different conditions (see Fig.5).

**Figure 4:**
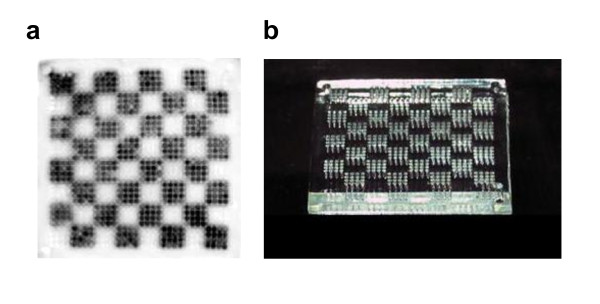
**MMV applications**. (a) Trial version of MMV handling. DNAs encoding the GFP gene charged in checker-patterned wells are PCR-amplified there and then transferred to another MMV where the transcription and translation experiments were performed, resulting in the expression of fluorescent GFP proteins. The variation in the fluorescence intensity came from rather weak controlling of transferred volumes (not completely eliminating lateral (side) transfer during the transfer by centrifugation), which should be conquered in future. (b) Photograph of a checker-patterned MMV. Well patterns of an MMV can be freely designed as described in Fig. 2d. For the sake of clarity, we chose a checker pattern to verify the transfer operation. Here, an MMV made of acrylamide gel is shown.

Following the methods described in Methods, the MMV was prepared and operated (see Fig. 2). MMVs were fabricated using the apparatus built in-house which is composed of DMD (Digital Multi-mirror Device) and others (Fig. 2a) [25,26]. We could form any type of vessels, filters, templates, and others made of either plastics or gel through light-induced polymerization by controlling the light pattern generated by DMD (Fig. 2b-d). MMVs made of gel (wet type) were first introduced here and used in combination with plastics MMVs (dry type) to fulfill multistep reactions as described in Methods. The basic operation of sample transfer from one to another MMV is an *aperture-to-aperture *way as shown in Fig. 3a and Fig. 3e. In order to demonstrate the feasibility of multistep reactions in the MMV, DNA encoding green fluorescent protein (GFP) was PCR-amplified using an MMV (see 'MMV operations' in Methods and Additional file 1). The amplified DNA molecules were transferred to another MMV, partly filled with a solution for the transcription/translation reaction, and then subjected to the reaction. The resulting checker pattern of fluorescent GFP proteins (see Fig. 4) confirmed the success of the parallel aperture-to-aperture transfer and the series of reactions: PCR, transcription, and translation which finally generate fluorescent GFPs (see 'Verification of the MMV transfer operation' in Methods).

Without using pipettes, each well of an MMV can be set to be differently conditioned ('multi-conditioning') by using various forms of sample-supplying MMVs (Fig. 3c and Fig. 3f). In other words, applying a sample to a particular well can be fulfilled by making the corresponding well chargeable as shown in Fig. 3f. On the other hand, solutions in each well can be transferred to a facing well in the opposing MMV in parallel, if necessary, separating magnet-bead-trapped molecules from the remaining solvent and solutes as shown in Fig.3d. Therefore, combining these operations, samples in each well can be independently manipulated at will without using pipettes, which is one of the most remarkable features of the MMV technology. Since handling of ~0.1 μL or less aliquots can not evade from the instant evaporation problem during the ejection of them from a microsyringe, this feature is valuable in addition to the rapid transfer. Besides, 1000-fold parallelism is another great problem in handling such a minute volume of solution even if robotics could be employed. This has been a common challenge for MEMS (mechano-electro micro system) and μTAS (micro-Total Analysis System) approaches [13-15,27,28], but yet not completely solved. Therefore, a direct transfer, aperture-to-aperture (Fig. 3e), system is an effective solution for this problem.

To make this system working, both wet and dry MMVs were necessary to be used compensatively: wet one is required for PCR and cell-cultivation while dry one can be used repeatedly for the other purposes such as exchange of solutions. This complementary usage of wet and dry MMVs enabled us to perform multistep reactions which contain molecule-amplification (PCR) reactions as shown later.

### Application to a crystallization condition test

**Figure 5:**
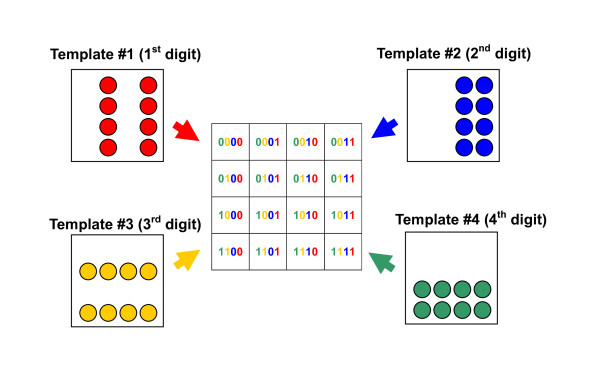
**2^N ^method**. Each well on an MMV can be addressed using the binary number. For example, in case of 16 wells-MMV (16 = 2^4^, N = 4), address-allotting becomes as shown in the center of this figure. If we regard each order (bit) corresponding to a different element (in this case, color), then 4 different (orthogonal) elements need to be prepared. If we take '1' as 'add' and '0' as 'not add', each binary addressing corresponds to directing which elements should be added there. So, the template way (see Fig. 3c) requires 4 different template plates as shown in this figure. By using these four templates, 16 different states can be prepared in an MMV.

**Figure 6:**
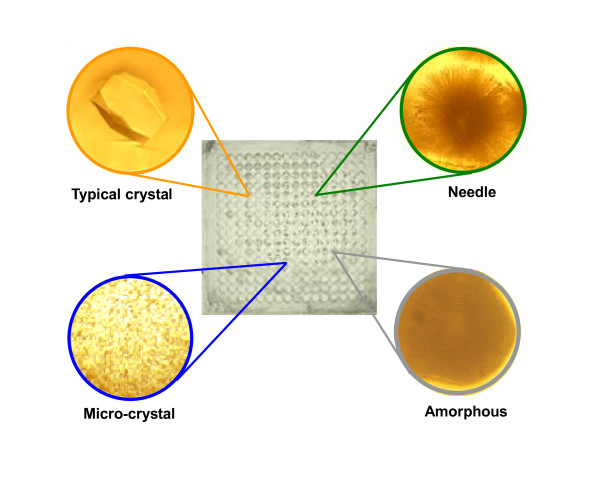
**Lysozyme crystallization**. In 256 different conditions, four distinctive types of crystals: typical, micro, needle-like and amorphous crystals were obtained. Some of the wells of an MMV are shown as inset (close-up images).

**Figure 7:**
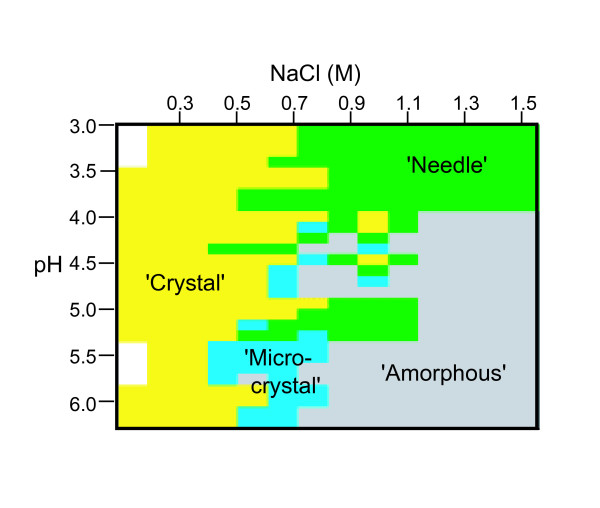
**Phase diagram of the crystallization of lysozyme**. Four distinctive types of crystals were obtained under the various ionic strength and pH conditions. Each state of crystallization is shown: yellow (typical crystal), green (needle-like crystal), blue (micro-crystal), gray (amorphous one), and white (undetected).

To generate multiple conditions (256 species), '2^N ^method' was employed using dry MMVs. In case of generating 256 different conditions, 8 species of MMV plates (2^N ^= 256; then N = 8) were used for this purpose (see Fig. 3f and Fig. 5). The transfer was carried out by spinning two face-to-face stacked MMV plates, a recipient MMV (bottom) and a donor MMV (top) (Fig. 3e). Using the MMV thus made, we could obtain four distinctive types of crystals: *typical, micro, needle-like *and *amorphous crystals *(Fig. 6) corresponding to the phase diagram in Fig. 7. This result informs us that the conditions of the lower NaCl concentrations (0.2~0.4 M) and the pH of around 4~5 were especially suitable for obtaining a large tetragonal crystalline and that the higher salt concentration (0.9~1.5 M) and the lower pH (pH 3~4) conditions generated the needle-like crystalline whereas the other conditions led to poor generation of crystallines. Such systematic information on crystallization conditions must be very useful for the physicochemical study of morphogenesis mechanism [29-32]. By applying this method, the cost-performance can be greatly improved due to the tiny scale (sub-μL) of this method and, otherwise unavailable experiments must be made possible (see '2^N ^Method' in Methods).

### Introduction and effectiveness of the wet MMV

**Figure 8:**
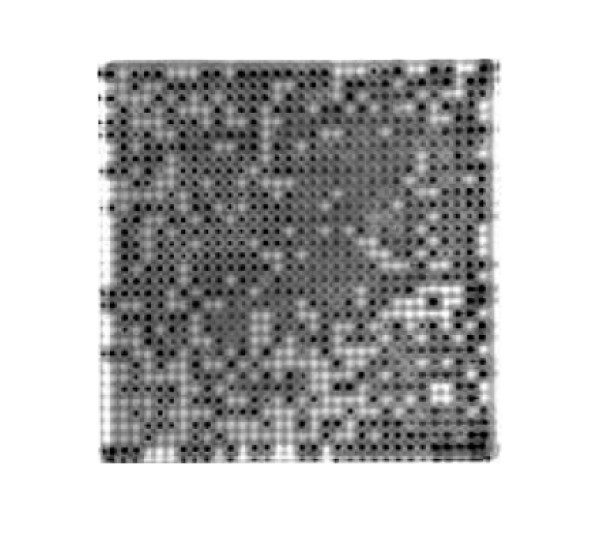
**MMV cultivation of *E. coli***. GFP-expressing cells of *E. coli *were detected by fluorescence with a fluoroimager (FITC mode): Dark wells indicate fluorescent colonies of *E. coli*.

In addition to the plastic MMV made of PDMS (polydimethylsiloxane) or acrylate resin as a dry type, we originally introduced a wet type MMV made of polyacrylamide gel. To validate wet MMVs used for cultivation, bacteria *E. coli *harboring green fluorescent protein (GFP) were subjected to a single cell culture (*i.e.*, a single cell per well (expectation value)). Overnight culture of the cells in the MMV resulted in a Poisson distribution as expected (Fig. 8). This means that we could obtain neatly arrayed colonies without depending on robotics such as Colony Picker [33], which can be easily manipulated in the following processes.

The power of wet MMVs was partly shown above in the success of the PCR-containing experiment (Fig. 4) since the dry MMVs could not have provided a successful PCR due to the drying-up during the PCR thermal cycle. Clearly, the wet gel must have protected the drying up effect of samples by serving as a reservoir against evaporation. Importantly, whether wet or dry MMVs are used, coating the surface of MMVs with BSA (1 mg/mL) was indispensable for successful reactions.

### Application of MMVs to multistep screening experiments

Two independent applications of MMVs were carried out: (a) selection of Amyloid β (Aβ)-binding peptides and (b) selection of cathepsin E inhibitory peptides.

**Figure 9:**
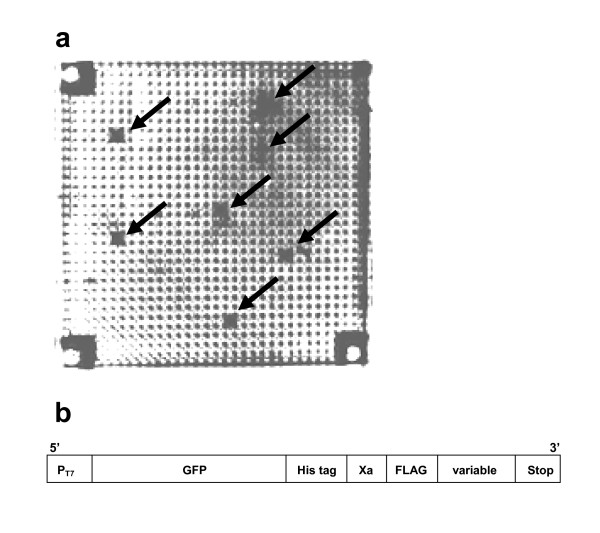
**Selection of Aβ-binding peptides performed in a 1000-wells MMV**. (a) Those wells which contain Aβ-binding peptides are indicated by an arrow (bright wells due to the fluorescence of the fused GFP). (b) DNA construct of the variable peptide-library. P_T7 _designates T7 promoter and franked by other regulatory sequences for transcription/translation of the gene. GFP means the green fluorescent protein gene. GFP is used for detection of peptide binding to Aβ on the magnetic beads. His tag and FLAG region are used for purification and detection of the protein. Xa is the recognition sequence for the restriction cutting of protease Xa. Variable region encodes the sequence of various peptides.

First, the selection of Aβ-binding peptides was performed, consisting of 5 consecutive steps of reaction: PCR-amplification of DNA, *in vitro *transcription, *in vitro *translation, binding of Aβ and Aβ-binding peptides, and monitoring the fluorescence of GFP (which is expressed as a protein fused with Aβ-binding peptides, thus serving as a marker) with three exchanges of MMV plates. The final result of these reactions is the Aβ-binding peptides as shown in Fig. 9 where those wells which contain GFP-fused peptides trapped by Aβ on a magnetic bead are illuminating (dark wells). Those wells where GFPs are expressed and halted (Aβ-binding peptides with a sufficient binding affinity with Aβ can be expected there) indicate the success of a series of reactions. The background brightness is caused by the reflection of the excitation beam on the surface of sample solutions. In this experiment, the peptide sequences could be obtained from the corresponding DNAs contained in the same well by PCR (data obtained are shown in Table 1).

**Table 1:** Selected Aβ binding peptides

Round/Clone No.	Nucleotide sequence (5'to 3')	Peptide sequence (10a.a.)
1/1	TGC ATT ATT ATT ATT TGG GAA CAC TCC TGC	CIIIIWEHSC
1/2	TGC ATT ATG TCC ATT CTC CTC ATT GTG TGC	CIMSILLIVC
1/3	TGC AAT AAT ACA GCG CCA AGT CAT AAT TGC	CNNTAPSHNC
2/1	TGC ATG TGG TGG ATT CCA ATT AAA CGT TGC	CMWWIPIKRC
2/2	TGC TGG GTA ATT TGG ATT GTG ATT ATG TGC	CWVIWIVIMC

**Figure 10:**
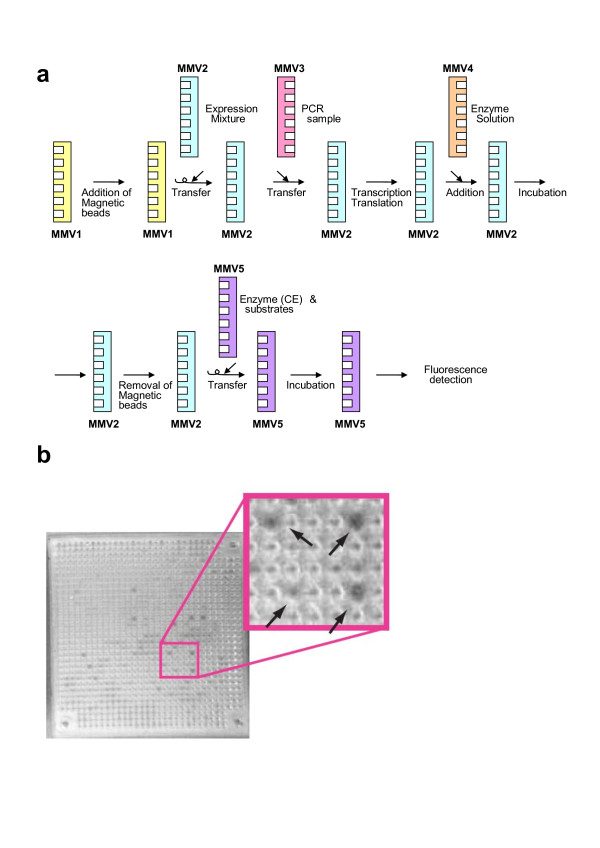
**Selection of cathepsin E (CE)-inhibitory peptides**. (a) An MMV plate plan adopted for the selection of CE-inhibitory peptides. First, magnetic beads were added to MMV1 and transferred to MMV2. MMV2 was changed with a cell-free transcription/translation solution. Independently, MMV3 was subjected to amplification of DNA (PCR reaction) and the products were transferred to MMV2. After transcription and translation reactions in MMV2, generated proteins were treated with restriction protease Xa, releasing peptides from the protein bound on the magnetic bead. After removal of magnetic beads, the supernatant was transferred to MMV5 which contains cathepsin E and its fluorogenic substrate. After incubation, the fluorescent product was monitored with a fluoroimager. The symbol 'arrow with a spiral' means that the preceding MMV is layered upside down as a donor MMV to the following one (recipient MMV). (b) Selection of CE-inhibitory peptides. In this case, dark wells contain CE-inhibitory peptides which block the CE enzyme reaction (generation of fluorescent products) and result in no production of fluorescent products. The arrows in inset indicate the inhibitory peptide-containing wells.

As another example of multistep reactions, selection of cathepsin E-inhibitory peptides was performed using MMV plates. For the multistep experiment, wet MMV plates were used for DNA amplification by PCR (starting with 50 molecules of template DNAs [34]), *in vitro *transcription/translation, restriction protease (Xa) digestion, and cathepsin E-inhibition assay (The MMV plate plan used is schematically shown in Fig. 10a). After all, the wells containing cathepsin E-inhibitory peptides were marked as a dark spot (Fig. 10b). The contents in those wells were further analyzed by DNA sequencing and the CE-inhibitory peptides were identified (see Additional file 2). Through this experiment, MMVs were again confirmed to be usable for multistep reactions including PCR.

## Conclusions

We have first introduced a novel concept microarray that can handle sub-μL scale bulk reactions, which enables 1000-fold parallel and multistep reactions. For this purpose we fabricated wet and dry *microarrays with manageable volumes *(MMVs). MMVs could be used for setting multi-different conditions and for culturing cells in an array mode (Fig. 6, Fig. 7 and Fig. 8). Especially, MMVs were powerful for *in vitro *selection experiments as shown regarding the selections of Aβ-binding peptides and CE inhibitory ones (Fig. 9 and Fig. 10). Conclusively, the MMV has two prominent merits: an ability to generate multiple conditions (1000-fold and more) and another ability to succeed well-specific products well-to-well (leading to multistep parallel reactions). Both were proven effective experimentally in this paper. We sometimes experienced mis-transfers in the aperture-to-aperture (well-to-well) mode because of side diffusion in-between two MMVs (partly discussed in Fig. 4). This problem must be solved controlling the quality of MMVs (Recently, this was verified by an experiment using MMVs made of PDMS (data not shown)). This technology is relatively free from the problems of evaporation (see Additional file 3) and adhesion losses owing to the direct transfer from well to well and the introduction of wet vessels made of gel. Since the volume of a solution is measured by the capacity of a well, there are some errors in the volume precision by around 10% or so, which is still permissible for the current studies. Moreover, this fault must be conquered by the improvement of the surface nature of dry MMVs (which sometimes work as a scale). Therefore, sub-μL volume, which is almost beyond the pipette operation, could be first manipulated quantitatively without depending on pipettes.

Finally, drawing a possible application of the MMV technology must be helpful to visualize the utility of this novel one. Namely, we will be able to fabricate a protein chip more conveniently and effectively than currently done. The procedures will be: i) make an oligonucleotide-array based on the conventional microarray technology, ii) trap cDNAs by hybridization at each cognitive oligonucleotide spot, iii) transfer each DNA parallelly to each corresponding well of an MMV by electrophoresis or diffusion (from spot to aperture), iv) do successive reactions in MMVs including PCR, *in vitro *transcription and *in vitro *translation on beads within a well, v) transfer the resultant peptides/proteins on beads on a chip surface and fix there by a tag sequence or else, completing a protein chip. This approach will drastically reduce the cost and labor to make arbitrarily designed protein chips. The protein chip thus constructed must be used for 'proteomic diagnosis' which aims to survey all biomarkers such as cancer, diabetes, neurodegenerative diseases, and others at once and quantitatively. To be encouraging enough, *in vitro *evolution utilizing the MMV itself (partly shown in Fig. 9 and Fig. 10) must be able to find such biomarkers.

## Methods

Tools and methods to construct the system of microarray-with-manageable-volumes (shortly, MMV) have been developed in this study, including an MMV generator, various types of MMV plates (made of gel or plastics with wells of different size and number), MMV containers for centrifugation and incubation and sample transfer/solution preparation methods.

### MMV-generator and MMV

MMVs were fabricated using a home-made apparatus, MMV generator which contains DMD (digital multi-mirror device) projector LVP-XD10 (Mitsubishi, Japan) with the optical system modified (Fig. 2a). This apparatus can project any light pattern under the direction of computer and thus can polymerize gel/plastics, of which reaction can be initiated by light irradiation, in an arbitrary shape (Fig. 2b). A typical dry MMV (1024 wells/inch^2^) is shown with the dimension of a well (Fig. 2c) and how to fabricate an MMV is depicted (Fig. 2d). Both types of MMVs, wet and dry, were developed using acrylamide aqueous solution and acrylate (pentaerythritol tri-acrylate; Aldrich, Germany), respectively. For the generation of polyacrylamide gel (wet MMV), 18% acrylamide solution (acrylamide:bis-acrylamide = 19:1) containing 0.27 mM riboflavin and 70% sucrose, was used. For acrylate resin (dry MMV), pentaerythritol tri-acrylate, containing 0.45% bis(2,4,6-trimethylbenzoil)-phenylphosfinoxide IRGACURE^®^819 (Ciba, Japan) and 0.05% 2-hydroxy-2-nethyl-1-phenyl-propane-1-on DAROCUR^®^1173 (Ciba, Japan), was used. After a brief light irradiation using mercury-lump VLT-X10P (Osram, Germany), unpolymerized solutions were immediately removed by jet-water (in case of wet MMV) or by spinning off (in case of dry MMV). The wet MMV was further subjected to a buffer-exchanging process to remove riboflavin, acrylamide monomers and sucrose and replace with an appropriate solution. Thus, the plate of a wet MMV was usually equilibrated with a reaction solution without large molecules such as peptides and DNAs. The swelling/shrinking effect of gels during these processes was kept carefully within control. The dry MMV was finally washed in a labware detergent 7-X ES PF (Dainippon Pharm. Co., Japan) with sonication.

### MMV operations

Due to the great number (~1000) and the smallness (~0.5 μL) of samples, one-by-one transfer of them is neither reasonable nor realistic. Therefore, we adopted the most direct approach for transferring samples from an MMV to an MMV by attaching two MMVs (donor MMV and recipient MMV) face-to-face and then precipitating by spinning (*aperture-to-aperture *transfer) and found this working. We further developed necessary techniques for handling MMVs: *charging, addition, and selective addition (filtering or template method) of samples, measuring up to a volume, mixing, incubation (cultivation or enzymatic reaction), PCR, separation into solid and liquid parts.*

#### a) Charging/Addition

Initial charging of sample solution into each well can be performed by spinning of a pool of solution placed on the top surface of MMV, resulting in filling all the wells with the solution and the remaining solution overflowed over the rim (see Fig. 3b). In case of adding another aliquot to the content of a well in an MMV, a transfer from the concerned MMV to an MMV having deeper wells (recipient MMV) was carried out in advance and then an additive transfer from an MMV (donor MMV) was made (see Fig. 3a). Further additions can be done until the recipient MMV is filled up.

(Development of a robotics is effective but not essential for most experiments.)

#### b) Selective addition (filtering/template method)

In those cases which require addition of a solution to particular wells, we can adopt either a filtering layer method or a template-like MMV one. The filtering layer placed between From- and To-MMVs enables the selective transfer from wells for which the filtering layer is open (penetrable). On the other hand, a template MMV can hold samples in those wells which were made open when the MMV was fabricated and thus can transfer samples selectively from those wells (see Fig. 3c).

#### c) Measuring up the volume

This can be done as an application of *addition *process (see above) if the size of the well of a donor-MMV is made unitary (of a fixed volume). N-times of addition make N units of volume input into a recipient-MMV.

#### d) Mixing

Although such a tiny volume as sub-μL is rather favorable for diffusion and thermal conductance, it is very unfavorable for mixing solutions with a vortex. For this purpose, we made a target molecule (peptide) bound on a magnetic bead to lift up and drop down by magnetic force. In other cases, stirring the solution with vigorous moving of small steel balls (~0.4 mm in diameter; Super micro-ball generously provided by Toyo Seiko Co., Japan) by magnetic force was also applied, of which effect was confirmed by diffusion of dye.

#### e) Incubation (cultivation/enzymatic reaction)/PCR

In case of culturing of bacteria, an MMV plate was equilibrated by soaking with LB broth or Davis medium prior to the culture and a container with a large space (more than 18 cm^3^) equipped with a water reservoir was selected. For a PCR reaction, the MMV plate was placed in a small container (5.4 cm^3^) firmly sealed to avoid an evaporation loss during incubation and held in a chamber thermally controlled (Mastercycler gradient, Eppendorf Co., Germany). PCR was carried out in a special device for the sake of effective heat conductance and minimum evaporation of water (see Additional file 1). The PCR program adopted was as follows: pre-heating, 94°C, 2 min; denaturing, 94°C, 0.5 min; annealing, 55°C, 1 min; extension, 72°C, 1 min (35 cycles); post extension, 72°C, 5 min.

#### f) Separation into a solid and a liquid parts

By attracting magnetic beads upward out of the liquid with a magnet, the solid (beads) and the liquid were easily separated (see Fig. 3d).

#### g) Image processing (fluorescence/optical light)

A newly made MMV was treated to equilibrate with a permeation solution (50 mM sodium acetate, 0.1 M NaCl, pH 4.5) containing 5 μM fluorogenic substrate of cathepsin E (CE) MOCAc-Gly-Lys-Pro-Ile-Ile-Phe-Phe-Arg-Leu-Lys(DnP)-D-Arg-NH_2 _(Code 3200-V, Peptide Institute, Inc., Osaka), and was half-filled with a CE reaction solution (permeation solution containing 5 μM substrate and 5 pmol CE additively). This reaction mixture was transferred to a translation MMV by centrifugation (1500 rpm, 20 s). The mixture was incubated at 37°C for 10 min with a shaking incubator Bioshaker V.BR-36 (TAITEC, Saitama). Then, the fluorescence of the product in the MMV was measured with a fluoroimager GelDoc XR (BioRad, USA) using 0.008% K_2_CrO_4 _aqueous solution as an excitation filter (320-340 nm) and a glass plate as an emission filter (<360 nm cut-off).

#### h) *In vitro *transcription and translation using an MMV

A wet MMV was treated to equilibrate with a permeation buffer for reverse transcription (13 mM magnesium acetate, 50 mM Hepes-KOH, 100 mM potassium glutamate, 20 mM creatine phosphate, 2 mM spermidine, 1 mM dithiothreitol, 2 mM ATP, 2 mM GTP, 1 mM CTP, 1 mM UTP, and 0.3 mM amino acid mix, pH 7.6). For *in vitro *transcription and translation, Wako pure system (Wako, Tokyo) was used and the reaction mixture was prepared following the manufacturer's instructions. Streptavidin-coated magnetic beads (TaKaRa, Kyoto) suspended in 80 μL (0.4 mg) and pre-washed repeatedly with 100 μL of water and the binding solution (TaKaRa, Kyoto) were combined and then added to every well of the MMV to half a well volume by manually pouring or controlled centrifugation. The MMV subjected to PCR (PCR MMV) was spun to precipitate solutions to the bottom before the following operation. In rare cases, some wells were readjusted to half a volume of the well with the PCR solution. Then, the PCR MMV was combined face-to-face to a translation MMV fitting the apertures in a correct phase with the 4 corners pinned and fixed. PCR solutions were transferred into each well of the translation MMV by centrifugation (1500 rpm, 20 s). The reaction solutions were stirred by moving magnetic beads up and down by attracting with a neodymium magnet (20 times). The mixtures were then incubated at 37°C for 2 h with further stirring at 20 min intervals as described above.

### Polymerase Chain Reaction (PCR) in MMV

A PCR mixture was prepared as recommended by manufacture's instruction (SpeedSTAR HS DNA polymerase TaKaRa, Kyoto), added optionally with an enzyme stabilizer (Lipidure BL-802 (NOF Corp. Tokyo)), and input into wells of a wet-type MMV which had been equilibrated in advance with PCR buffer containing PCR components except template DNA, Taq polymerase, and primers. The MMV was placed within a PCR container which was hand-made from silicon rubber and stainless-steel sheets (see Additional file 1). The container was placed in a thermal cycler and PCR amplification was performed with a program (pre-denaturation, 94°C, 2 min; denaturation, 94°C, 1 min; annealing, 55°C, 1 min; extension, 72°C, 1 min (35 cycles); and post-extension, 72°C, 5 min). The MMV plate was removed from the container and the content was recovered after centrifugation. The DNA amplification with this system was confirmed by gel-electrophoresis and silver staining (see Additional file 1).

### Verification of the MMV transfer operation

To demonstrate the effectiveness of the well-to-well transfer and successive reactions, we constructed and tried a model experiment. A checker-patterned MMV was filled with a PCR solution containing 2 fmole/μL of DNA coding GFP as templates, respectively. SpeedSTAR polymerase (TaKaRa, Kyoto) was used for a rapid amplification and the PCR program was utilized as follows: pre-denature, 94°C, 2 min; denature, 94°C, 20 s; annealing and extension, 1 min (25 cycles); and post-extension, 72°C, 2 min. After PCR procedures (see 'MMV operations' in Methods), the contents of the MMV were transferred to another MMV which was partly filled with a cell-free transcription/translation solution (see 'MMV operations' in Methods). Then, this MMV was incubated at 37°C for 1 h. The fluorescent image was monitored with a fluoroimager and the checkered fluorescent image was obtained. The consistence between the PCR well-pattern and the fluorescent image of GFP verified the fidelity of MMV transfer operations (see Fig. 4).

### 2^N ^Method

In the case of micro-arrays which have a 2^N ^× 2^N ^square well-pattern ("N" designates a natural number), its whole diversity (2^2N^) can be generated by employing "2N" kinds of quite different (orthogonal) elements. "2N" kinds of elements can be easily prepared by using "2N" sheets of filters (by considering the symmetrical nature of filters, "N" is sufficient) which have rather simple well patterns. In the case of "N" = 2 (see Fig. 5) for example, 2 sheets of filter are sufficient to be prepared and each filter can be used in two ways, working virtually as four template plates. Four kinds of elements are transferred into a recipient microarray by centrifuge. Finally, all of the 16 wells of the microarray would have different constituents from each other. Each well can be uniquely assigned by the binary number. If we regard the digit '1' as 'exist' and '0' as 'non-exist' and if we consider the difference of the order in the number corresponds to the difference of elements in the actual experiment, then the well assigned 1001 (9 in the decimal number) should contain the elements corresponding to the elements #1 and #4 (#1-#4 corresponds to the MSB, 2nd MSB, 3rd MSB and LSB). Here, we utilized a 256-well micro-array ("N" = 4) for examining condition on re-crystallization of lysozyme (see Fig. 2f where we can confirm that the pairs of T0/T4, T1/T5, T2/T6, and T3/T7 are symmetrical).

### Selection of Aβ aptamers

The DNA construct for *in vitro *transcription/translation (see Fig. 9b) was amplified using the following primers in the PCR reaction: P1, 5'GATCCCGCGAAATTAATACGACTC ACTATA3'; and P2, 5'GGCTCGCGAATACTGCGAAGGAGTGAGATC3'. A part of the translation mixture and the magnetic beads were removed from the translation MMV using a magnet. On the other hand, Aβ peptides were trapped on magnetic beads as follows: an aliquot containing 50 μL Magnotex SA particle (TaKaRa, Kyoto) was mixed with biotin-labeled Aβ peptide solution (5 μg/50-500 μL) in a binding buffer. After incubation for 20 min at room temperature, the beads were washed and re-suspended with 500 μL PBS buffer (pH 7.0). For each selection round, 2 mg of beads, which have approximately 500 pmoles of bound Aβ (1-42), was used. Magnetic beads-Aβ conjugates were inputted into a fresh MMV to make each well containing 0.050 pmoles (0.5 μL) of the conjugate. Then, the conjugate suspension was spun down into a translation MMV and the solution in the wells was mixed by moving the magnetic beads up and down with a magnet. The suspension was incubated at 37°C for 1 h with several 20 min-interval mixings. With fixing the magnetic beads with a magnet on the bottom of well, the solution was transferred to a fresh MMV by centrifugation. The fluorescence of GFP bound on beads was monitored with a fluoroimager. After selection of Aβ-binding peptides by fluorescence intensity, the separated supernatants were used as a template solution for PCR for the next round of selection and DNA sequencing to confirm the Aβ-binding peptides.

### Library construction and selections

#### a) Library construction

Combinatorial DNA and IVV (*in vitro *virus)-peptide libraries were constructed according to the previously reported method of YLBS [35] and cDNA display [36].

#### b) Preparation of CE-immobilized beads used for selection

Purified cathepsin-E (CE) was immobilized on NHS-activated sepharose beads (GE Healthcare, USA) by using the amine coupling chemicals to form a chemically stable amide bond in accordance with the manufacturer's instructions. The enzyme-coupled beads were stored in 100 mM phosphate buffer (pH 7.4) at 4°C until further use. The coupling efficiency was calculated by comparing the absorbance of uncoupled enzyme with that of free enzyme.

#### c) Affinity-based selection

The IVV-peptide library in 100 μL of Selection buffer (50 mM Tris-HCl, 100 mM NaCl, 5 mM MgCl_2_, pH 7.2) was mixed with 5 μL of CE-immobilized beads and incubated at 25°C for 30 min. The beads were washed with 200 μL of Selection buffer, Washing buffer-1 (50 mM Tris-HCl, 0.5 M NaCl, pH 7.2) and Washing buffer-2 (50 mM Tris-HCl, 1 M NaCl, pH 7.2) in accordance with the protocol of washing repeat count, which varied along the selection round, i.e., 2:2:1 in Round 1; 2:2:2 in Round 2; 3:3:3 in Round 3. Finally, the beads were suspended in 200 μL of Elution buffer-1 (50 mM Tris-HCl, 1 M NaCl, 10 mM MgCl_2_, pH 7.2) and incubated at 37°C for 5 min. After centrifugation (1500 rpm, 1 min), the beads were washed again with Elution buffer-1. The combined supernatant was stored (Sup1). The beads were suspended with Elution buffer-2 (50 mM Tris-HCl, 2 M NaCl, 10 mM MgCl_2_, pH 7.2) and incubated at 95°C for 5 min. After incubation, the beads were removed and the supernatant was stored (Sup 2). The IVVs in Sup1 and Sup 2 were purified with a Bio-Spin column (Bio-Rad Laboratories, USA).

#### d) Function-based selection

The DNA library resulting from Round 3 of the affinity-based selection was inserted in the IVV-SF-link DNA construct as a variable region [36]. The IVVs were prepared and incubated as described in affinity-based selection. The protocol of washing repeat count with Selection buffer, Washing buffer-1 and Washing buffer-2 was carried out according to the selection round program (F1 to F9) as follows: 3:3:3 in F1, 4:5:3 in F2, 5:5:5 in F3, 5:7:5 in F4, 5:7:7 in F5, 5:10:7 in F6, 5:10:10 in F7, 5:15:10 in F8, and 5:15:15 in F9.

### Sample preparation

#### a) GFP-expressing *Escherichia coli*

The buffer components in a gel-MMV plate (1 × SSC) were exchanged with LB broth and the affluent solution in wells was removed by centrifugation. Overnight culture of *E. coli *cells harbouring green fluorescence protein (GFP) was diluted with LB broth to the concentration of one cell per well (0.5 μL) and then put into wells of an MMV by centrifugation. The MMV was layered on a wet tissue paper in a petri dish and subjected to the incubation at 37°C. The fluorescence of GFP expressed in *E. coli *cells was monitored with a fluoroimager, Molecular Imager FX (Bio-Rad Laboratories, USA).

#### b) A DNA library for cathepsin E-inhibitory peptides

**Figure 11:**
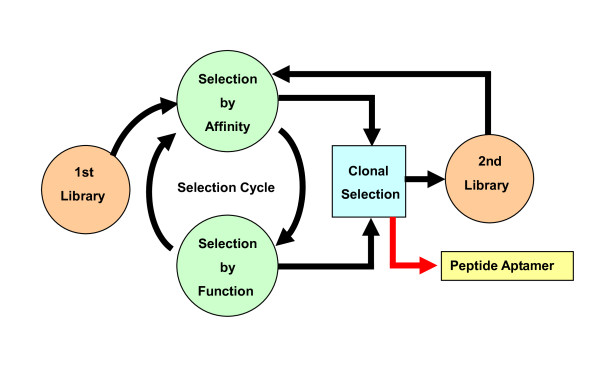
**Schematic presentation of library-based evolutionary molecular engineering**. The first library which consists of arbitrarily collected members is subjected to the selection by affinity and then by function in the selection cycle. The selected molecules are processed to the clone-by-clone selection. Based on the information obtained from these selections, the second library is constructed. Furthermore, the second library is advanced to the selection cycle until identifying target peptides. MMVs can be utilized in the clonal selection. For details, see [34] in the text.

A DNA library was constructed, of which DNA encodes a different species of a cathepsin E-binding peptide (8-28 amino acids in size) which is derived from the preceding *in vitro selection *experiment [36] (see ' Library construction and selection' and Fig. 11).

#### c) Lysozyme solutions

Lysozyme provided in lyophilized powder (protein content, ~95%; ~50,000 units/mg protein (Sigma-Aldrich, Germany)) was dissolved in distilled water (250 mg/mL) using a vortex mixer with a special care so as not to leave visible flocs and then spun down. The supernatant was used for the crystallization experiment.

### Generation of combinatorial conditions (2^N ^method)

We applied the 2^N ^method (see '2^N ^Method' and Fig. 5) using a 256-well MMV to generating 256 species of conditions aimed for crystallization of lysozyme. The ionic strength and pH were modulated using the following NaCl and sodium acetate-HCl buffers, respectively. Each template MMV of T0~T7 was charged with 0.1 μL of 2 M NaCl (T0), 3 M NaCl (T1), 4 M NaCl (T2), 5 M NaCl (T3), pH 3 (T4), pH 4 (T5), pH 5 (T6), and pH 7 (T7), respectively. To make the final solution volume equal in each well, the complementary volume of water was added to those wells which were not charged with any of T0~T7 solutions, using the complementary templates T0~T7 (well/non-well relations inverted). Finally, a lysozyme solution (250 mg/mL) was added (0.1 μL). The MMV was covered with a piece of transparent adhesive tape Titer Stick (Wakenyaku, Japan) to prevent evaporation and was kept at 20°C for several days. Each well was monitored by an inverted microscope IM (Olympus, Tokyo).

### Multistep operations of MMVs

#### a) *In vitro *selection of Aβ-binding peptides

The whole procedure is described in detail above, in which the construct and the selection of a peptide-fused GFP is described (see Fig. 9). Peptides were expressed by successive reactions of PCR, *in vitro *transcription and *in vitro *translation as written above. Then, Aβ protein (Aβ42) linked to a magnetic bead was added by an MMV-transfer (*i.e.*, transfer from an MMV to another MMV by centrifugation). Those peptides bound to Aβ were separated from the remaining unbound ones by the next MMV-transfer (see 'MMV operation'), washed with a buffer (10 mM Tris-HCl, 1 mM EDTA, 1 M NaCl, 0.1% NaN_2_), and then subjected to the fluorescence monitoring using fluoroimager Molecular Imager FX (Bio-Rad Laboratories, USA) at the excitation wavelength of 488 nm.

#### b) *In vitro selection *of cathepsin E-inhibitory peptides

A set of peptides which have a cathepsin E-inhibition activity selected in the preceding study [34] were further screened using MMVs (see Fig. 10). The DNA molecules encoding those peptides were diluted and input in an MMV (~50 copies per well) and then subjected to PCR, *in vitro *transcription/translation, and protease Xa digestion. Then, the magnetic beads were removed from the MMV. The remaining solutions were used to detect the inhibition activity of the peptide in each well. The volume of the reaction mixtures were, if too much, reduced to around a half-well volume by sucking with filter paper. The measurement of the inhibition activity in each well was carried out as described in Methods (Image processing).

## Authors' contributions

YK and TT contributed equally. YK performed most of the finalizing experiments for optimizing and improving MMVs and obtaining the data and jointly wrote and edited this paper. TT carried out the most of the initial and advancing stage of this study. KK and MSa made the application experiment (CE inhibitor selection and Aβ binding peptide selection, respectively). HU fabricated the MMV generation machine. MSu helped directing the experiment and jointly edited the paper. YH efforted to begin and promote this study and provided essential discussions. KN designed, directed and wrote and edited this paper. All authors read and approved the final manuscript.

## Supplementary Material

Additional file 1 **Additional Figure 1 - PCR reaction using an MMV**. (a) PCR device specialized for the microarray. Upper cover and Bottom container were fabricated of stainless steel sheet and silicon rubber. The microarray in the container was heat-treated via the heat block of PCR instrument. To avoid the leakage of vapor from the container, Upper cover was pressed with a high temperature lid which prevented the vapor from condensing on the cover. (b) PCR product (780 bp) was recovered by centrifuge and analyzed by polyacrylamide gel electrophoresis and silver staining. DNA templates were amplified from 50 molecules (lane 1) and 10 molecules (lane 2) per well, respectively. Lane "M" shows marker DNA bands (upper: 850 bp, lower: 750 bp). Click here for file

Additional file 2 **Additional Table 1 - Cathepsin E-inhibitory peptides obtained by the MMV method**. A list of peptides selected by the MMV method is given. Click here for file

Additional file 3 **Additional Figure 2 - Evaporation rate curve**. Evaporation rates were measured with an MMV filled with water placed on ice (square) or on the lab bench (triangle) under the conditions of room temperature (~15°C) and humidity (47% and 58%, respectively). Click here for file

## References

[B1] Schena M Shalon D Davis RW Brown PO Quantitative monitoring of gene expression patterns with a complementary DNA microarray Science 1995 270 467 470 10.1126/science.270.5235.467 7569999

[B2] Schulze A Downward J Navigating gene expression using microarrays - a technology review Nature Cell Biol 2001 3 E190 E195 10.1038/35087138 11483980

[B3] Saizieu A Certa U Warrington J Gray C Keck W Mous J Bacterial transcript imaging by hybridization of total RNA to oligonucleotide arrays Nat Biotechnol 1998 16 45 48 10.1038/nbt0198-45 9447592

[B4] Lipshuts RJ Fodor SPA Gingeras TR Lockhart DJ High density synthetic oligonucleotide arrays Nat Genet 1999 21 20 24 10.1038/4447 9915496

[B5] Mitchell P A perspective on protein microarrays Nat Biotechnol 2002 20 225 229 10.1038/nbt0302-225 11875416

[B6] Emili AQ Cagney G Large-scale functional analysis using peptide or protein arrays Nat Biotechnol 2000 18 393 397 10.1038/74442 10748518

[B7] Zhu H Bilgin M Bangham R Hall D Casamayor A Bertone P Lan N Jansen R Bidlingmaier S Houfek T Mitchell T Miller P Dean RA Gerstein M Snyder M Global analysis of protein activities using proteome chips Science 2001 293 2101 2105 10.1126/science.1062191 11474067

[B8] Winkler DF Campbell WD The spot technique: synthesis and screening of peptide macroarrays on cellulose membrane Methods Mol Biol 2008 494 47 70 full_text 18726568 10.1007/978-1-59745-419-3_4

[B9] Jermutus L Honegger A Schwesinger F Hanes J Plückthun A Tailoring *in vitro *evolution of protein affinity or stability Proc Natl Acad Sci 2001 98 75 80 10.1073/pnas.011311398 11134506 PMC14547

[B10] Yuan L Kurek I English J Keenan R Laboratory-directed protein evolution Microbiol Mol Biol Rev 2005 69 373 392 10.1128/MMBR.69.3.373-392.2005 16148303 PMC1197809

[B11] Biyani M Husimi Y Nemoto N Solid-phase translation and RNA-protein fusion: a novel approach for folding quality control and direct immobilization of proteins using anchored mRNA Nucleic Acids Res 2006 34 e140 10.1093/nar/gkl771 17062621 PMC1635333

[B12] Naimuddin M Kitamura K Kinoshita Y Honda-Takahashi Y Murakami M Ito M Yamamoto K Hanada K Husimi Y Nishigaki K Selection-by-function: efficient enrichment of cathepsin E inhibitors from a DNA library J Mol Recognit 2007 20 58 68 10.1002/jmr.812 17173335

[B13] Nakane J Broemeling D Donaldson R Marziali1 A Willis TD O'Keefe M Davis RW A method for parallel, automated, thermal cycling of submicroliter samples Genome Res 2001 11 441 447 10.1101/gr.GR1644R 11230168 PMC311064

[B14] Yang J Liu Y Rauch CB Stevens RL Liu RH Lenigk R Grodzinski P High sensitivity PCR assay in plastic micro reactors Lab Chip 2002 2 179 187 10.1039/b208405h 15100807

[B15] Marcus JS Anderson WF Quake SR Parallel picoliter RT-PCR assays using microfluidics Anal Chem 2006 78 956 958 10.1021/ac0513865 16448074

[B16] Gerlach A Knebel1 G Guber AE Heckele M Herrmann D Muslija A Sshaller TH Microfabrication of single-use plastic microfluidic devices for high-throughput screening and DNA analysis Microsystem Technol 2002 7 265 268 10.1007/s005420100114

[B17] Auroux PA Iossifidis D Reyes DR Manz A Micro Total Analysis Systems. 2. Analytical Standard Operations and Applications Anal Chem 2002 74 2637 2652 10.1021/ac020239t 12090654

[B18] Rogers Y-H Venter JC Massively parallel sequencing Nature 2005 437 326 327 10.1038/437326a 16163333

[B19] Nemoto N Biyani M Hosoi Y Ichiki T On-chip Evolution: Overcoming of fluctuation in analysis of a single molecule activity in a pico-liter well Biophysics 2006 46 S330

[B20] Barbulovic-Nad I Lucente M Sun Y Zhang M Wheeler AR Bussmann M Bio-microarray fabrication techniques--a review Crit Rev Biotechnol 2006 26 237 259 10.1080/07388550600978358 17095434

[B21] Xu Q Lam KS Protein and chemical microarrays-powerful tools for proteomics J Biomed Biotechnol 2003 2003 257 266 10.1155/S1110724303209220 14688413 PMC521501

[B22] Donay JL Mathieu D Fernandes P Prégermain C Bruel P Wargnier A Casin I Weill FX Lagrange PH Herrmann1 JL Evaluation of the automated Phoenix system for potential routine use in the clinical microbiology laboratory J Clin Microbiol 2004 42 1542 1546 10.1128/JCM.42.4.1542-1546.2004 15071001 PMC387561

[B23] Vincentelli R Canaana S Offanta J Cambillaua C Bignon C Automated expression and solubility screening of His-tagged proteins in 96-well format Anal Biochem 2005 346 77 84 10.1016/j.ab.2005.07.039 16168382

[B24] Sundberg SA High-throughput and ultra-high-throughput screening: solution- and cell-based approaches Curr Opn Biotech 2000 11 47 53 10.1016/S0958-1669(99)00051-8 10679349

[B25] Sun C Fang N Wu DM Zhang X Projection micro-stereolithography using digital micro-mirror dynamic mask Sens Actuat A 2005 121 113 120 10.1016/j.sna.2004.12.011

[B26] Sato N Hasegawa Y Uchida H Fabrication and fluorescence measurement system for microarray using Digital Micromirror Device IEIC Technic Rep 2006 106 1 6

[B27] Nagai H Murakami Y Yokoyama K Tamiya E High-throughput PCR in silicon based microchamber array Biosensors & Bioelectronics 2001 16 1015 1019 10.1016/s0956-5663(01)00248-2 11679283

[B28] Lee SJ Lee SY Micro total analysis system (μTAS) in biotechnology Appl Microbiol Biotechnol 2004 64 289 299 10.1007/s00253-003-1515-0 14714150

[B29] Alderton G Fevold HL Direct crystallization of lysozyme from egg white and some crystalline salts of lysozyme J Biol Chem 1946 164 1 5 20989461

[B30] Pusey ML Snyder RS Naumann R Protein crystal growth. Growth kinetics for tetragonal lysozyme crystals J Biol Chem 1986 261 6524 6529 3700405

[B31] Ataka M Tanaka S The growth of large single crystals of lysozyme Biopolymers 1986 25 337 350 10.1002/bip.360250213 3955194

[B32] Saridakis E Chayen NE Towards a 'universal' nucleant for protein crystallization Trends Biotechnol 2009 27 99 107 10.1016/j.tibtech.2008.10.008 19110330

[B33] Jones P Watson A Davies M Stubbings S Integration of image analysis and robotics into a fully automated colony picking and plate handling system Nucleic Acids Res 1992 20 4599 4606 10.1093/nar/20.17.4599 1408762 PMC334190

[B34] Kitamura K Yoshida C Kinoshita Y Kadowaki T Takahashi Y Tayama T Kawakubo T Naimuddin M Salimullah M Nemoto N Hanada K Husimi Y Yamamoto K Nishigaki K Development of systemic *in vitro *evolution and its application to generation of peptide aptamer-based inhibitors of cathepsin E J Mol Biol 2009 387 1186 1198 10.1016/j.jmb.2008.12.028 19150354

[B35] Kitamura K Kinoshita Y Narasaki S Nemoto N Husimi Y Nishigaki K Construction of block-shuffled libraries of DNA for evolutionary protein engineering: Y-ligation-based block shuffling Protein Eng 2002 15 843 853 10.1093/protein/15.10.843 12468719

[B36] Tabuchi I Soramoto S Nemoto N Husimi Y An *in vitro *DNA virus for *in vitro *protein evolution FEBS Lett 2001 508 309 312 10.1016/S0014-5793(01)03075-7 11728441

