# Supplementary figures and images for: Novel concept microarray enabling PCR and multistep reactions through pipette-free aperture-to-aperture parallel transfer
Source: BMC Biotechnol. 2010 Oct 6;10:71. doi: 10.1186/1472-6750-10-71 (PMC2959086; doi:10.1186/1472-6750-10-71)

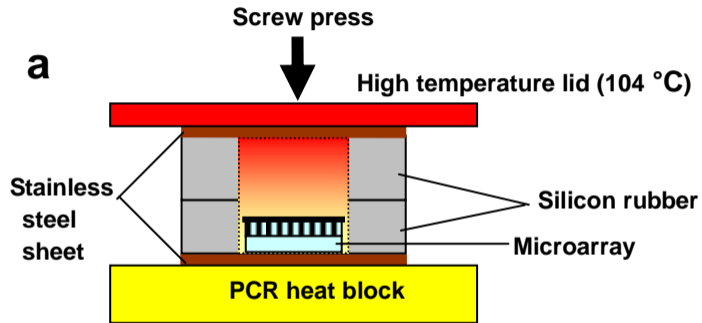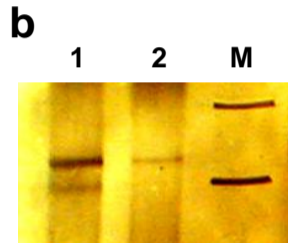

Supplement: Additional file 1 — Additional Figure 1 - PCR reaction using an MMV. (a) PCR device specialized for the microarray. Upper cover and Bottom container were fabricated of stainless steel sheet and silicon rubber. The microarray in the container was heat-treated via the heat block of PCR instrument. To avoid the leakage of vapor from the container, Upper cover was pressed with a high temperature lid which prevented the vapor from condensing on the cover. (b) PCR product (780 bp) was recovered by centrifuge and analyzed by polyacrylamide gel electrophoresis and silver staining. DNA templates were amplified from 50 molecules (lane 1) and 10 molecules (lane 2) per well, respectively. Lane "M" shows marker DNA bands (upper: 850 bp, lower: 750 bp). [file 1472-6750-10-71-S1.PDF]

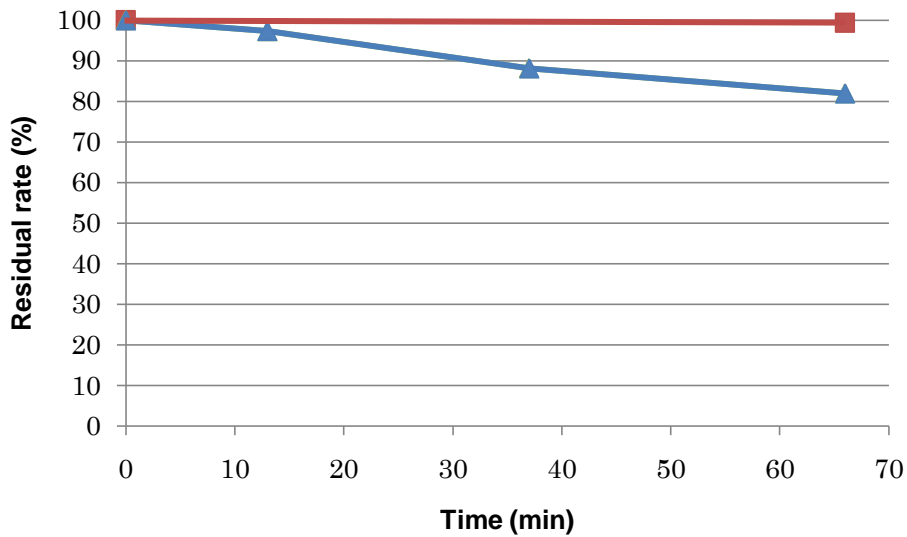

Supplement: Additional file 3 — Additional Figure 2 - Evaporation rate curve. Evaporation rates were measured with an MMV filled with water placed on ice (square) or on the lab bench (triangle) under the conditions of room temperature (~15°C) and humidity (47% and 58%, respectively). [file 1472-6750-10-71-S3.PDF]
