# Supplementary material for: Novel concept microarray enabling PCR and multistep reactions through pipette-free aperture-to-aperture parallel transfer
Source: BMC Biotechnol. 2010 Oct 6;10:71. doi: 10.1186/1472-6750-10-71 (PMC2959086; doi:10.1186/1472-6750-10-71)
Supplement: Additional file 2 — Additional Table 1 - Cathepsin E-inhibitory peptides obtained by the MMV method. A list of peptides selected by the MMV method is given. [file 1472-6750-10-71-S2.PDF]

Additional Table 1 Cathepsin E-inhibitory peptides obtained by the MMV method [†]

Serial no.	Amino acid sequence (N→C)	Size (a.a.)	Inhibition Activity(%) I_{sy} [‡]	IC_{50} (%)	Comments
101	SCGG IIII SCIA	12	45.2	220	*K*i=5 nM:*K*d=62 nM Non-competitive
102	NDDK IIII CCII	12	43.9	280	*K*i=14 nM Competitive
103	NYKD SCIG	8	31.1	680	*K*i=3 nM Non-competitive
104	NDDK IIII PTIF GG	14	39.9	260	—
105	IIII SCIG DDGH QKKK K	17	34.9	240	Frameshift at the center onward
106	SGLL FRLK GG	10	20.6	320	—
107	GGRP IIII GG	10	14.5	4600	*K*d=150 nM

[†] Taken from [34] (Kitamura *et al*. *J*. *Mol*. *Biol*. **387**, 1186-1198 (2009)).

[‡] I_{sy} represents the activity of a synthetic peptide.
